# Neonatal mortality in dogs: Prognostic value of Doppler ductus venosus waveform evaluation - Preliminary results

**DOI:** 10.14202/vetworld.2016.356-360

**Published:** 2016-04-07

**Authors:** Gabriele Barella, Stefano Faverzani, Massimo Faustini, Debora Groppetti, Alessandro Pecile

**Affiliations:** Department of Veterinary Medicine, State University of Milan, Via Celoria 10; 20133; Milan, Italy

**Keywords:** dog, Doppler, ductus venosus, neonatal mortality, ultrasound

## Abstract

**Aim:**

To define the prognostic value of Doppler ultrasonographic morphology of ductus venosus (DV) waveform on canine neonatal mortality.

**Materials and Methods:**

Fifty-four healthy pregnant bitches underwent fetal ultrasonographic assessment. The DV waveforms were classified as diphasic (dDVw) or triphasic (tDVw) and compared with neonatal mortality.

**Results:**

Ninety-three fetuses were evaluated. Twenty fetuses belonged to litters with neonatal mortality, in which tDVw was observed. Seven fetuses belonged to litters without neonatal mortality, in which tDVw was observed. Fifty-eight fetuses belonged to litters without neonatal mortality, in which only dDVw was observed. Eight fetuses belonged to litters with neonatal mortality, in which only dDVw was observed. The correlation between tDVw and neonatal mortality was statistically significant (odds ratio [OR], 20.7; p<0.0001). Considering only pregnancies with one or two fetuses with the same DV waveform: Two fetuses with tDVw belonged to litters with neonatal mortality; 1 foetus with tDVw belonged to litter without neonatal mortality and 26 fetuses showed dDVw without neonatal mortality. The correlation between tDVw and neonatal mortality even in litters up to two pups was statistically significant (OR, 88.3; p=0.01).

**Conclusion:**

Echo-Doppler assessment of DV is feasible in canine fetuses, and the presence tDVw seems to be related to neonatal mortality.

## Introduction

The ultrasonographic evaluation of ductus venosus (DV) hemodynamic plays an important role in human medicine since it is considered a relevant indicator of fetal well-being [1,2]. The DV is a small trumpet-shaped vessel of a few millimeters length and width, originating from the umbilical vein and reaching the caudal vena cava near the confluence of the hepatic veins, gradually reducing its diameter. Several studies reported ultrasonographic evaluation of DV blood flow in fetal lambs [3,4] and in dogs [5]. According to human literature the ultrasonographic Doppler waveforms of DV are classified as monophasic, diphasic or triphasic (dDVw or tDVw) [6], while only two different types of waveforms have been described in canine fetuses (diphasic or triphasic) [5].

In humans, the presence of a triphasic waveform in the DV (reverse a-wave which corresponds to a deflection during atrial contraction) is considered a strong predictor of neonatal mortality independently of gestational age [7].

This preliminary study aims to evaluate the Doppler ultrasonographic waveforms of the DV and to verify the predictive ability of the tDVw in neonatal mortality in dogs.

## Materials and Methods

### Ethical approval

The present study has been carried out on privately owned dogs and include no any clinical trials. The present study was performed in accordance with the ethical guidelines of animal welfare committee and informed consent was obtained from each owner.**Data collection**

Fifty-four clinically healthy private owned pregnant bitches (primiparous or multiparous) were recruited. The population was composed by mixed and purebred dogs such as American Staffordshire, Bernese Mountain Dog, Boston Terrier, Chihuahua, Dogo Argentino, Dogue de Bordeaux, English Bulldog, French Bulldog, Golden Retriever, Great Dane, Jack Russel Terrier, Labrador Retriever, Pinscher, Rhodesian Ridgeback, Shetland Sheepdog, and Yorkshire Terrier. All dogs were considered healthy based on physical examination, blood cell count, serum chemistry, and urinalysis results. No clinical pathological evidence of disease was recorded through any pregnancy. Neonatal mortality defined stillborn pups and those died within 6 h of life. The present study was performed in accordance with the ethical guidelines of animal welfare committee and informed consent was obtained from each owner.

To deduce the LH peak and the optimal time for mating, all bitches underwent an accurate reproductive cycle monitoring by vaginal cytology and plasma progesterone measurement [8,9].

Plasma progesterone concentration was determined using a quantitative test based on ELFA technique (Enzyme Linked Fluorescent Assay; MiniVidas, Biomerieux). Assay principle combines an enzyme immunoassay competition method with a final fluorescent detection [10]. Bitches were included in the study if the initial progesterone sample at proestrus was <2 ng/mL. The first day on which the serum progesterone was ≥2 ng/mL was regarded as the LH peak [8,9]. Gestational age was calculated from the estimated LH peak (Day 0). The number of born puppies, the date and type of delivery were recorded.

All dogs underwent ultrasonographic evaluation once during pregnancy. The bitches were evaluated in dorsal or lateral recumbency (without any pharmacological restraint), clipping the hair from the costal arch to the inguinal region, and applying a conductive gel to the skin. Ultrasonographic diagnosis of pregnancy and fetal monitoring were performed using two-dimensional and Doppler ultrasound (Esaote MyLab 70, Genua, Italy) with a high multi-frequency linear probe (7.5-13 MHz). Waveform in the DV was measured in only two fetuses [11,12] during each examination, except in cases of singleton pregnancy). The fetuses selected for measurements were the ones that were located most cranially and caudally within the uterus, but all recognizable fetuses were evaluated for viability based on ultrasonographic evaluations of the fetal movements [13] and heartbeat [13,14]. The DV was identified following the intra-abdominal course of the umbilical vein until a trumpet-shaped vessel was seen entering the caudal vena cava, as previously described by Kiresud *et al*. [1]. The color aliasing artifact was used for correctly identify the DV (this color phenomenon is responsible for the “mosaic” appearance of the DV).

**Figure 1:**
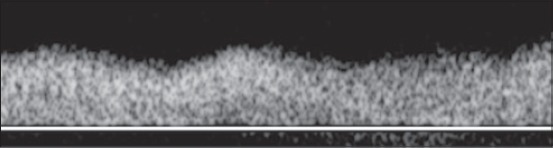
Pulsed-wave Doppler image of monophasic waveform of a portal vein as an example.

**Figure 2:**
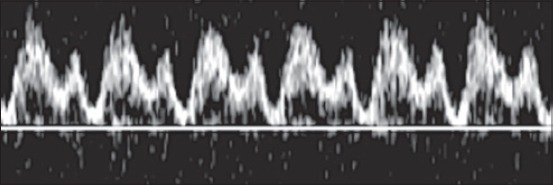
Pulsed-wave Doppler image of a diphasic waveform of a ductus venosus.

**Figure 3:**
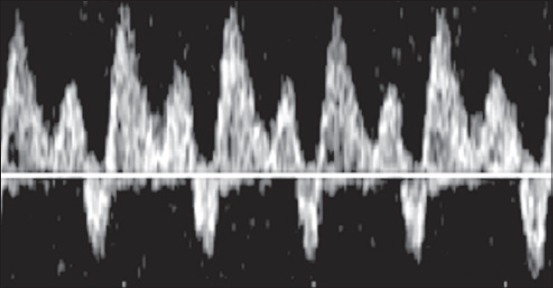
Pulsed-wave Doppler image of a triphasic waveform of a ductus venosus.

Pulsed-wave Doppler was then applied to acquire the waveforms. The angle of the pulsed Doppler sample volume was set for angle-dependent measurements between 20° and 60°. To avoid the signal from nearby vessels, the sample volume was set to 1-2 mm. The Doppler and color filters were set at 40-100 Hz, and the velocity was set at 8-12 waveforms in one screen image. A sequence of at least three successive and symmetric waves was required to classify them as monophasic, diphasic or triphasic. The DV waveform was considered monophasic (Figure-1) if there were no modulations other than those attributable to breathing; diphasic (Figure-2) if all the modulations were in the same direction and none of them reached the baseline; triphasic (Figure-3) if two modulations were seen on one side of the baseline and one was seen on the opposite side of the baseline. Images were collected and downloaded to a computer.

### Statistical analysis

Odds ratios (OR) were calculated, with 95% confidence intervals (CI), to compare the chance of neonatal mortality between litters with one or more tDVw and litters without tDVw. The test sensibility (Se) and specificity (Sp) were also calculated as the relative positive predicting value (PPV) and negative predicting value (NPV).

## Results

Fifty-four bitches were included in the study. The bitches ranged in age from 1 to 7 years (2.8±1.2 years), and weighed from 4 to 50 kg (16.6±12.3 kg). Ninety-three fetuses out of the 213 born pups were evaluated (43.7%).

Sixty puppies were born by elective cesarean section from 25 bitches, and 153 puppies were born by vaginal parturition from 29 bitches. All bitches with vaginal delivery were monitored during labor. No fetal ingestion occurred. During gestational period, the pups number was not determined. However, all bitches were evaluated again after labor with ultrasound to confirm that each bitch delivered every pups that were in uterus. Litter sizes ranged from 1 to 10 (mean value of 4±3 puppies). Neonatal mortality (within 6 h of birth) was 9.8% (21 out of 213) with nine dead necropsied pups affected by: One pup had severe chest wall deformation; one pup was anasarca; three pups had bilateral renal agenesis; four pups had a premature appearance. The remaining 192 pups (90.1%) were healthy. All bitches underwent ultrasonographic evaluation once from 36 to 66 days of pregnancy (mean 53.5±8 days). No bitch had an abortion. Considering that the earlier Doppler measurement was performed at day 36 of gestation, we can assume that no measured foetus was reabsorbed. The DV was identified in all the 93 fetuses considered and the waveform was classified as diphasic (66 waveforms out of 93; 71%) or triphasic (27 waveforms out of 93; 29%). No monophasic DV waveform was detected.

Distribution of dDVw and tDVw respect to neonatal mortality is showed in Table-1. The OR showed a statistically significant correlation between the presence of a tDVw and neonatal mortality (OR, 20.7; 95% CI, 6.7-64.4; p<0.0001). Se and Sp were 71.4% and 89.2% with PPV 74.1% and NPV 87.9%, respectively. If we consider the pregnancies with only one or two fetuses, 35 fetuses were evaluated (15 singleton from 15 bitches and 20 fetuses from 10 bitches). Among the 10 pregnancies with two fetuses, only in seven the fetuses presented the same DV waveform (either tDVw or dDVw). The distribution of dDVw and tDVw respect to neonatal mortality in pregnancies with one foetus (n=15) and in pregnancies (n=7) with two fetuses with the same DV waveform (n=29 fetuses) is represented in Table-1. The OR showed a statistically significant correlation between the presence of a tDVw and neonatal mortality (OR, 88.3; 95% CI, 2.8-2791.9; p=0.01). The Se and Sp were 100% and 96.3% with PPV 66.67% and NPV 100%, respectively.

**Table-1:** Table summarizing population data.

ID	Breed	Weight (kg)	Age (year)	GA (day)	Waveform	Type of parturition	Neonatal mortality	Number of pups
1	DogoArgentino	33	3	50	dDV	C	No	2
2	Labrador retriever	25	2.5	43	dDV	C	No	2
3	Bouledogue	8	3	63	tDV	C	Yes	6
4	Staffordshire bull t.	18	4	52	dDV	N	No	6
5	Shetland	9	4.5	56	dDV/tDV	N	Yes	5
6	DogoArgentino	35.5	2	57	dDV	C	No	2
7	Am. Staffordshire t.	34.5	3	49	dDV	N	No	7
8	Great Dane	50	5	58	dDV/tDV	N	No	10
9	Chihuahua	4	2	57	dDV	N	No	3
10	Yorkshire	5.5	3	51	tDV	N	Yes	4
11	Bulldog	21	1.5	49	dDV	C	No	1
12	Bulldog	19	2	60	dDV	C	No	1
13	Boston t.	12.5	4	64	dDV	C	No	2
14	Chihuahua	3.5	2.5	58	dDV/tDV	C	Yes	2
15	Pinscher	9	2	46	dDV	N	No	1
16	Pug	9.5	2.5	40	dDV	N	Yes	3
17	Jack russel t.	8.5	2.5	64	tDV	N	Yes	1
18	Bulldog	8	2	55	dDV	N	Yes	5
19	Rhodesian Ridgeback	33	2	38	dDV	N	No	10
20	Chihuahua	6	2	47	dDV/tDV	C	No	4
21	Bulldog	21.5	3	58	dDV	C	No	1
22	Bouledogue	8	3	60	dDV	C	No	5
23	Boston t.	9	5	66	dDV	C	No	1
24	Am. Pit bull t.	28	2	36	dDV	N	No	10
25	Cross breed	9	3	53	tDV	N	Yes	4
26	Bouledogue	7.5	1	39	dDV	N	No	1
27	Staffordshire bull t.	17	3	59	dDV	N	No	6
28	Pinscher	9.5	2	43	tDV	C	No	1
29	Bouledogue	9	2.5	61	tDV	C	Yes	5
30	Pug	8	5	63	dDV	C	No	2
31	Chihuahua	5	2.5	38	dDV	C	No	4
32	DogoArgentino	31.5	2	54	dDV	N	No	9
33	Bouledogue	9.5	4	60	dDV/tDV	N	No	7
34	Am. Staffordshire t.	28.5	2	45	dDV	N	No	7
35	Benese Mountain dog	30.5	3.5	39	dDV	N	No	9
36	Yorkshire	5	4.5	60	tDV	N	Yes	4
37	Golden r.	30	2	52	dDV	N	No	9
38	Chihuahua	4	2	55	dDV/tDV	N	Yes	2
39	Bouledogue	10.5	5	47	tDV	C	Yes	7
40	Pinscher	9.5	1	60	dDV	N	No	2
41	Shetland	7	5	49	tDV	N	Yes	4
42	Rhodesian Ridgeback	38	2	53	dDV	N	No	8
43	Pug	13	2	62	dDV	C	No	1
44	Bulldog	20	1	56	dDV	C	No	1
45	DogoArgentino	30.5	2.5	50	dDV	C	No	2
46	Jack russel t.	6	3	60	tDV	N	Yes	1
47	Boston t.	8	2	66	dDV	C	No	1
48	Dogue de Bordeaux	38	3	50	dDV	N	No	9
49	Great dane	47.5	4	60	dDV	N	No	5
50	Chihuahua	4,5	2	52	dDV/tDV	C	Yes	2
51	Boston t.	7	6,5	64	tDV	C	No	3
52	Yorkshire	8.5	2	51	dDV	C	No	1
53	Boston t.	10	1	48	dDV	N	No	1
54	Bulldog	17.5	2	63	dDV	C	No	1

dDV=Diphasic ductus venosus waveform, tDV=Triphasi cductus venosus waveform, C=Cesarean section, N=Natural vaginal delivery, GA=Gestational age

## Discussion

**Figure 4:**
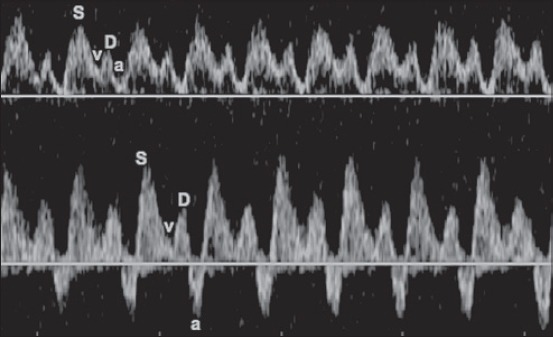
At the top a diphasic ductus venosus waveform of a canine fetus; at the bottom a triphasic ductus venosus waveform of another fetus. S=Ventricular systole, v=Atrial diastole, D=Ventricular diastole, a=Atrial systole.

The bloodflow waveform of DV is commonly used in hemodynamic evaluation of human fetuses [15]. It reflects the cardiac cycle: the S-wave corresponds to the ventricular systole, the v-wave corresponds to the atrial diastole, the D-wave corresponds to the ventricular diastole and the a-wave corresponds to the atrial systole (Figure-4). The abnormalities of DV waveform such as reverse flow during the atrial systole, that is triphasic waveform, are considered strong predictors of stillbirth [7]. The a-wave reflects the capacity of the heart to accommodate venous return that depends on venous volume, cardiac function (relaxation, compliance and contractility) and downstream arterial bloodflow resistance. The presence of a maintained dDVw in fetuses predicts an intact survival [7].

Our study shows the feasibility to access the hemodynamic of canine fetal DV during pregnancy. However, Doppler recording requires some training and patience to reach a reliable level of skill [16]. The DV was identified in all the 93 fetuses considered; the waveform was diphasic in 71% of subjects while the remaining 29% of fetuses presented a tDVw. As expected a monophasic DV waveform was not observed in this study; indeed, this wave depends on breathing movements that cannot be present in the foetus.

Neonatal mortality was significantly associated with reverse DV a-wave (tDVw): Litters in which tDVw was recorded had more chance (almost 21 times more) to present neonatal mortality (one or more dead pups per litter) than those with only dDVw.

The inability to identify the single foetus among other littermates (both in subsequent ultrasonographic evaluations through pregnancy) is a common limit occurring in fetal evaluation of polytocous species. The evaluation of only 2 fetuses in our work is obviously a biasing factor of our results; however in every obstetric study concerning the ultrasonographic evaluation of canine fetuses only 2 pups are normally evaluated [11,12] and in many studies even just one [17-19]. In our study, we evaluated two fetuses to be sure to evaluate 2 different subjects. To our knowledge the only way we can evaluate every subjects would be during cesarean section, using an intraoperative ultrasound technique. Statistical correlation between tDVw and neonatal mortality resulted in an acceptable Sp (89.2%) and a poor Se (71.4%). This finding could depend on decision to evaluate only two fetuses per litter. Regarding the eight fetuses belonged to litters with neonatal mortality in which only dDVw was recorded (Table-1), we hypothesized that there might be other puppies not valued in the same litter presenting tDVw.

Further, seven fetuses belonged to litters without neonatal mortality in which at least a tDVw was observed (Table-1). We cannot explain this finding, but we noticed that all these fetuses belonged to bitches that underwent cesarean section. However, our study does not describe, in any way, the influence of Cesarean section on neonatal mortality.

It is known that pregnancies with one or two fetuses are predisposed to higher neonatal mortality, and so we decided to analyze data referring only to these subjects. The OR calculated in singleton or twin concordant fetuses showed 88 times higher risk of death in fetuses with tDVw compared to those with dDVw, and Se and Sp were very high (respectively 100% and 96.3%).

We remark that tDVw is not the cause of death of these pups, it can be considered as a parameter of fetal disvitality as an expression of a circulatory distress.

In humans, the abnormalities of DV bloodflow have been associated to some pathological conditions such as aneuploidy, congenital heart diseases, fetal acidosis, hypoxemia, intrauterine growth restriction, oligohydramnios, and fetal anemia [20,21]. As mentioned above, it was not possible to correlate DV bloodflow characteristics to specific diseases we found in our sample. Further, four pups died within 6 h after birth (two French bouledouge, one Boston terrier) had bilateral renal agenesis. A relationship between renal agenesis and tDVw has never been postulated. However, in human fetuses with severe urinary tract malformations, a cardiorenal syndrome characterized by a bilateral ventricular hypertrophy (caused by an increased afterload) was described by Merz *et al*. [22] and it is known that a reverse DV a-wave (tDVw) can be a consequence of an increase afterload [7].

## Conclusions

Our study does not correlate at all the presence of tDVw and a specific cause of death, but rather the presence of tDVw seems to be predicting the risk of neonatal mortality in the litter. When reverse DV a-wave is found, an accurate pregnancy monitoring and perinatal assistance should be scheduled. Although these preliminary results are encouraging, further studies are needed to deepen the relationship between fetal DV abnormalities and specific pathologies in dogs.

## Authors’ Contributions

GB, SF, and DG designed the study project and retrieved all data during the study period. GB, SF, DG and AP performed the clinical procedures. MF, GB, SF, and DG analyzed the data. The paper was written by GB, SF and DG. All authors read and approved the final manuscript.
